# Media use pattern as an indicator of mental health in the COVID-19 pandemic: Dataset from India

**DOI:** 10.1016/j.dib.2021.106722

**Published:** 2021-01-09

**Authors:** Mrinal Mukherjee, Chanchal Maity, Somdutta Chatterjee

**Affiliations:** a The West Bengal University of Teachers’ Training, Education Planning and Administration (WBUTTEPA), India; b Bankura University, India

**Keywords:** COVID-19 pandemic, Media use pattern, Mental well-being, Mental anxiety, Media amplification

## Abstract

This article presents data on the media use pattern of respondents with different degrees of mental well-being and mental anxiety in the context of the COVID-19 pandemic. We collected data on demographic variables, patterns of media engagement, and levels of mental well-being and mental anxiety among the Indian adult population in the COVID-19 era. A web-based cross-sectional online survey was conducted to obtain data on two main aspects in the context of COVID-19: mental well-being and mental anxiety and engagement with the media, both television and other social media channels. Using respondent-driven convenient sampling method, 426 Indian adults (age ≥ 18 years) residing in the country responded to the survey. The survey was conducted 3 weeks after the nationwide lockdown was enforced between April 16 and 22, 2020. Besides providing the risk messages about the disease outbreak, the media channels provided sensational coverage of it that might have amplified the risk perception of the public; thus, media use pattern may be a strong indicator of the impact of COVID-19 on the mental health of an individual. Therefore, this dataset could serve as a reference base for in-depth studies on the association between media amplification of a pandemic and the mental health status of the common public in the context of social disaster.

## Specifications Table

**Table:** 

Subject	Psychiatry and Mental Health
Specific subject area	Association between mental well-being and mental anxiety and media engagement pattern
Type of data	Table
How data were acquired	The data were collected through an online survey and converted into .xlsx format for further analysis by SPSS 16.0 (IBM corporation).
Data format	Raw Analyzed Filtered
Parameters for data collection	The target population was Indian nationals, who were fluent in English language, aged 18 years or above, and residing in India.
Description of data collection	A web-based cross-sectional online survey was conducted on the two main dimensions, namely mental well-being and mental anxiety among the Indian public and their engagement, in terms of the amount of time spent, with the media. Respondent-driven convenient sampling was applied 3 weeks after the enforcement of a nationwide lockdown between April 16 and 22, 2020. All participants gave the necessary information and granted informed consent before starting the survey.
Data source location	Information was collected from adult resident citizens of India, irrespective of the state (province) of residence, caste, creed, religion, and sex; the researchers did not consciously exclude any social group***.*** The respondents represented metropolitan, urban municipal, and rural areas.
Data accessibility	http://dx.doi.org/10.17632/sktz4xv4vh.4

## Value of the Data

• This data set was collected in the first phase of the lockdown when the respondents were under home quarantine; this dataset can be instrumental for studies aiming to compare the sustained effects of a lockdown on the dimensions of mental health in longitudinal studies.
• The intricacies of the relationship between media amplification of a said event and mental well-being and mental anxiety can be explored for any social disaster at any point of time using this data set.
• Researchers, policy makers, and stakeholders may benefit from such a dataset in understanding and designing mental health policies from the public health perspective in the Indian context.
• This datasets may be instrumental for researchers who wish to compare the indicators of mental health (e.g., degree of intensity) and media engagement in the COVID-19 pandemic and post-pandemic periods.

## 1 Data Description

On March 11, 2020, the WHO declared that the COVID-19 outbreak was a “pandemic” [1]. In India, a nationwide lockdown was declared on March 25, 2020, and the quarantine has had unprecedented effects on the population. The people started becoming heavily dependent on the media [2], including online and print media, and media amplification of the pandemic situation had increased the stress and anxiety levels [3], [4], [5]. Therefore, the researchers conducted a web-based cross-sectional survey to assess the impact of media engagement with indicators of mental health, namely mental well-being and mental anxiety in the COVID-19 pandemic era.

Table 1 shows the distribution of the respondents for each demographic category: sex, age, residence, and educational qualification. As the exposure to social environment varies between men, women, and intersex individuals, sex was considered a variable in this study. No intersex person responded to the survey.

**Table 1:** Descriptive statistics of demographic characteristics (*N* = 426) and percentage-based representation of target population.

	Sample representatives		

Category	N	Percentage	Target population (in million), % of representation in the sample
**Sex**			
Male	236	55	1.95, .012
Female	190	45	0.55, .035
**Age**			
Young	306	72	2.0, .015
Middle aged	108	25	0.43, .025
Old aged	12	03	0.07, .017
**Residence**			
Rural	90	21	0.72, .012
Urban Municipal	190	45	0.95, .020
Metropolitan City	146	34	0.83, .017
**Educational qualification**			
UG	113	27	2.0, .005
PG	239	56	0.28, .085
PG Above	74	17	0.02, .370

UG, undergraduate; PG, post-graduate; PG-above, MPhil/PhD/any higher degree

Age-based categorization was carried out considering that the psychosocial perception of individuals varies according to age. The respondents aged between 18 and 38 years were considered young, between 39 and 60 years, as middle aged, and 60 and above, as old-aged people. The age group of 18 to 38 was considered young from the perspective of general health status. The life insurance policy regulators in India endorse people aged 18–35 years under the sound health category as they are less susceptible to diseases than those aged below or above this age limit. In our study, we extended the upper limit to 38 years. In India, people aged above 60 years are considered senior citizens; hence, those aged 60 years and above were categorized under the old-aged group. All respondents between 39 and 59 were categorized under the middle-aged group.

In many social studies, the place of residence is found to influence the psycho-social reflections of individuals. We considered the local socio-cultural context of the place of residence and instead of following the standard rural–urban binary, three residence categories were considered: metropolitan, urban municipal, and rural. In India, the rural societal texture shows much homogeneity while the small cities are quite different from metro cities in terms of lifestyle and culture. The respondents living in small cities are culturally influenced by both urban and rural factors and thus are categorized under the urban municipal category. We received responses from people living in all three residence categories.

The level of education is an influential factor in shaping the psychosocial maturity and responses of individuals. As this convenient sample comprised educated sections of the population, three educational levels of tertiary education were considered: Under graduate (UG), post-graduate (PG), and MPhil/PhD/any higher degree (PG-above).

According to the survey conducted jointly by the UN delegation in India, the Ministry Youth Affairs and Sports, the Government of India, and the United Nations Development Programme (2019), approximately 300 million active social media users having either a WhatsApp or Facebook account in India [6]. Another survey conducted in August 2019 by NapoleonCat reported that almost 7% of social media users belonged to the teenage group (13–17 years) [7]. Therefore, 93% of the population included in the study was above 18 years of age. According to the last census of India (2011), only 8.5% of the Indian population has attained educational qualifications at graduate level or above, and only 10.6% of the population has basic knowledge of English language (as the first, second, or third language) [8]. Considering the minimum age limit of 18 years and effective English knowledge are pre-conditions for participation in this survey, the target population was approximately 2.5 million. Among social media users, nearly 78% are male while only 22% are female, and as per age-wise distribution, 80% are young, 17% are middle aged, and 3% are old aged [7]. The Internet and Mobile Association of India (2014) reported that the percentages of rural, urban municipal, and metropolitan (the top four metro cities were considered) social media users were 29%, 38%, and 33%, respectively [9]. As there is no substantial evidence on percentage-wise distribution of social media users on the basis of educational qualification, the average enrollment ratio in higher education was considered the reference to calculate the approximate percentage distribution, resulting in 80%, 11%, and 1% of the sample having UG, PG, and PG-above qualifications, respectively [10]. The percentage-wise distribution of the sample for each demographic category is shown in Table 1.

The dataset showed heavily skewed demographic distribution, with overrepresentation in female, middle aged, urban municipal, PG, and PG-above categories. Such representation could be attributed to the limitation of the data collection method. The data were collected through channels such as Facebook and WhatsApp and hence may not be show even distribution in all categories. Considering the reality of the lockdown, the researchers could not avoid this kind of bias in the data collection process. The study is also limited as the researchers had to sometimes rely on data published by non-governmental agencies to analyze the target population.

Table 2 shows the percentage-wise distribution of respondents against each item on media use pattern, with options. This part of the survey covered the hour-wise engagement with media channels, preferences based on trustworthiness, and perceptions of the role of social media channels, particularly in the context of the pandemic.

**Table 2:** Percentage analysis of media use pattern (*N* = 426).

No.	Description	Category	Category wise respondents (n)	Percentage
1	How many hours a day do you spend on social media channels?	<5 h	69	16.2
		5–9 h	291	68.3
		>9 h	66	15.5
2	How many hours a day do you spend watching news on the TV?	<2 h	267	62.7
		2–3 h	116	27.2
		>3 h	43	10.1
3	On which type of media channel do you rely the most for authentic news or information?	Social media	104	24.4
		Television	238	55.9
		Others	84	19.7
4	What type of media channel according to you is the most dependable during the COVID-19 crisis?	Social media	95	22.3
		Television	266	62.4
		Others	65	15.3
5	Through which media channels do you keep yourself updated about the ongoing COVID-19 crisis?	Social media	134	31.4
		Television	238	55.9
		Others	54	12.7
6	Do you think that social media channels spread more fake news than other media?	Yes	346	81.2
		No	80	18.8
7	Do you verify the information that you receive on social media channels?	Always	157	36.9
		Mostly	209	49.1
		Rarely	52	12.2
		Never	8	1.88
8	Do you post information on COVID-19 to social media channels without verifying it?	Yes	36	8.5
		No	390	91.5
9	Do you refer to the WHO & ICMR websites for understanding and maintaining COVID-19-related hygiene?	Yes	332	77.9
		No	94	22.1
10	Do you think that despite some negative implications, social media channels played a significant role during the COVID-19 crisis?	Yes	382	89.7
		No	44	10.3

The respondents’ engagement with the media in hours per day (combining item nos. 1 & 2 in Table 2) was denoted as “Media Use” and the scores obtained by the respondents on the mental well-being and anxiety scales were denoted as “Mental Well-being” and “Mental Anxiety” scores. These three major variables were further divided into the “high”, “medium”, and “low” categories to understand the response pattern. The Media Use and Mental Well-being scores were categorized using percentile distribution, and the Mental Anxiety scores were categorized using the predetermined criteria mentioned by the scale developers. The categorization method is outlined in the subsequent section. The percentage-wise distribution of the respondents for each variable is depicted in Table 3.

**Table 3:** Percentage wise distribution of the study sample (*N* = 426) in high, medium, and low categories of each of variable.

Media use (N, %)			Mental well-being (N, %)			Mental anxiety (N, %)		
High	Medium	Low	High	Medium	Low	High	Medium	Low
129, 30.3%	144, 33.8%	153, 35.9%	121, 28.4%	195, 45.8%	110, 25.8%	28, 6.6%	49, 11.5%	349, 81.9%

Table 4 shows the percentage-wise distribution of the respondents into high, medium, and low categories of media use, mental well-being, and mental anxiety against the demographic variables of sex, age, residence, and educational qualifications. The table also highlights the pattern of media use, mental well-being, and mental anxiety for each demographic characteristic and the trends of influence of the demographic features on these variables.

**Table 4:** Demography-based category distribution against major variables.

	Media use (N, % within)			Mental Well-being(N, % within)			Mental Anxiety(N, % within)		

Demographic characteristic	High	Med	Low	High	Med	Low	High	Med	Low
**Sex**									
Male	81, 34.3	77, 32.6	78, 33.1	68, 28.8	112, 47.5	56, 23.7	16, 6.8	24, 10.2	196, 83.1
Female	48, 25.3	67, 35.3	75, 39.5	53, 27.9	83, 43.7	54, 28.4	12, 6.3	25, 13.2	153, 80.5
**Age**									
Young	105, 34.3	105, 34.3	96, 31.4	68, 22.2	150, 49.0	88, 28.8	23, 7.5	36, 11.8	247, 80.7
Middle aged	23, 21.3	35, 32.4	50, 46.3	49, 45.4	39, 36.1	20, 18.5	04, 3.7	12, 11.1	92, 85.2
Old aged	1, 8.3	4, 33.3	7, 58.3	04, 33.3	06, 50.0	02, 16.7	01, 8.3	01, 8.3	10, 83.3
**Residence**									
Rural	40, 44.4	22, 24.4	28, 31.1	23, 25.6	44, 48.9	23, 25.6	12, 13.3	17, 18.9	61, 67.8
Urban Municipal	56, 29.5	66, 34.7	68, 35.8	51, 26.8	81, 42.6	58, 30.5	06, 3.2	21, 11.1	163, 85.6
Metropolitan City	33, 22.6	56, 38.4	57, 39.0	47, 32.2	70, 47.9	29, 19.9	10, 6.8	11, 7.5	125, 85.6
**Educational qualification**									
UG	47, 41.6	36, 31.9	30, 26.5	24, 21.2	58, 51.3	31, 27.4	03, 2.7	09, 8.0	101, 89.4
PG	66, 27.6	86, 36.0	87, 36.4	70, 29.3	109, 45.6	60, 25.1	19, 7.9	26, 10.9	194, 81.2
PG- Above	16, 21.6	22, 29.7	36, 48.6	27, 36.5	28, 37.8	19, 25.7	06, 8.1	14, 18.9	54, 73.0

## 2 Experimental Design, Materials and Methods

The survey was conducted through an online platform 3 weeks after the enforcement of a nationwide lockdown from April 16 to 22, 2020. The period chosen for this survey was the very beginning of the spread of COVID-19 infection in India when the nation was in the first phase of lockdown. Indiscriminate circulation of both authentic facts as well as manufactured information about disease intensity and virulence of the pathogen and subsequent spread of exaggerated information were found to be causal factors for mental anxiety and deteriorating mental well-being in previous epidemic events. Research findings on the Ebola outbreak in Africa showed a similar impact of media amplifications observed among the US public, where a high degree of uncertainty regarding the threat of Ebola led to high stress and anxiety [11].

The data were collected through a Google form, using respondent-driven convenient sampling method [12,13]. A survey link was first uploaded on WhatsApp and Facebook and sent to the contacts of the investigators. Apart from providing personal response, the respondents were also requested to forward the link to the other people, so the link was circulated beyond the first point of contact. In every case, on accessing the link, the respondent was auto directed to the information about the study and informed consent form. The questionnaire was divided into four parts. In the first part, the respondents were required to fill in their respective demographic details. In the second part, the respondents were directed to a set of 10 items on media use. The third and fourth parts included items on mental well-being and anxiety, respectively.

The survey link was distributed on social media channels and only the persons with adequate knowledge of the English language and WhatsApp and Facebook accounts could participate in the study. It was an online survey conducted during the lockdown period. Though the typical sampling frame was absent, based on previous survey reports in the literature, the approximate target population was 2.5 million. Since random sampling is not feasible for such a large sample, respondent-driven convenient sampling was used as an alternative. The data obtained from a non-probability sampling design also has potential for use in inferential statistics; our design allowed the respondents to forward the survey link to their contacts to reduce the sampling bias, reduced the dependence on the initial convenience sample, and allowed us to reach a wide range of the population [13,14].

To assess the media use pattern during the lockdown period, 10 structured validated items regarding the information and news about COVID-19 were used, e.g. the perspectives of different sources like social media channels, electronic media, print media, and online news sources, etc. Ten items were used to capture the pattern of engagement with the media during the lockdown. Based on the total length of engagement with the media in a day (combining item nos. 1 & 2 in Table 2), the respondents were categorized into three groups: High (>9 h), medium (8–9 h), and low (<8 h). This categorization was based on the percentile distribution of the length of media engagement. The 25^th^ and 75^th^ percentiles were termed as “low” and “high” media use patterns, corresponding to 7 and 10 hours, respectively. The scores between these two cut-off percentiles were placed under the “medium” category of media use.

The Warwick-Edinburgh Mental Well-Being Scale [15] was used to collect data about mental health during home isolation in the lockdown period. It was a five-point Likert type scale with 14 items, with scores ranging from “none of the time” = 1 to “all the time” = 5, for items such as “I've been feeling confident”. The summated score of each respondent was used as the mental well-being score, categorized into high (score, >56), medium (score, 47–56), and low (score, <47) mental well-being following the percentile distribution of the score of the respondents, as mentioned in the categorization of media use.

Beck's Anxiety Inventory (BAI) Scale [16] was used to collect information on the mental anxiety level of the respondents during this crisis period. It consists of a four-point Likert scale with 21 items, anchored at “Not at all” = 0, “Mildly – but it didn't bother me much” = 1, “Moderately – it wasn't pleasant at times” = 2, and “Severely – it bothered me a lot” = 3. The summated score of each respondent was used as the mental anxiety score. The mental anxiety levels were also categorized in the BAI scale itself as high (score, >35), medium (score, 22–35), and low (score, <22).

Statistical analysis was performed using SPSS 16.0 (IBM Corporation). Descriptive statistics included percentage analysis that was applied to estimate the demographic and category-wise distribution (high, medium, and low) of the media use pattern, mental well-being, and mental anxiety of the respondents.

## Ethics Statement

Authors are here confirming that informed consent was obtained from all the participants and they were assured confidentiality of their personal information.

## CRediT Author Statement

**Mrinal Mukherjee**: Conceptualization, Methodology, Data Curation, Data Analysis, Resources, Supervision, Writing - Original Draft, Review & Editing; **Chanchal Maity**: Methodology, Software Operation, Validation, Data Analysis, Investigation, Resources, Data Curation, Writing - Original draft, Review & Editing; **Somdutta Chatterjee:** Methodology, Data Analysis, Investigation, Resources, Writing - Review & Editing.

## Declaration of Competing Interest

The authors declare that they have no known competing financial interests or personal relationships which have, or could be perceived to have, influenced the work reported in this article.
